# Tocilizumab in chronic active antibody-mediated rejection: rationale and protocol of an in-progress randomized controlled open-label multi-center trial (INTERCEPT study)

**DOI:** 10.1186/s13063-024-08020-0

**Published:** 2024-03-22

**Authors:** Lillian Streichart, Marie Felldin, Jana Ekberg, Lars Mjörnstedt, Per Lindnér, Annette Lennerling, Verena Bröcker, Johan Mölne, Jan Holgersson, Kristien Daenen, Lars Wennberg, Tomas Lorant, Seema Baid-Agrawal

**Affiliations:** 1 Transplant Institute, Sahlgrenska University Hospital, Institute of Clinical Sciences, Sahlgrenska Academy at University of Gothenburg, 413 45 Gothenburg, Sweden; 2 Department of Pathology, Sahlgrenska University Hospital, Gothenburg, Sweden; 3 Department of Laboratory Medicine, Institute of Biomedicine, University of Gothenburg and Department of Clinical Immunology and Transfusion Medicine, Sahlgrenska University Hospital, Gothenburg, Sweden; 4 Department of Nephrology, Sahlgrenska University Hospital, Gothenburg, Sweden; 5 Department of Transplantation Surgery, Karolinska University Hospital, Stockholm, Sweden; 6 Section of Transplantation Surgery, Department of Surgical Sciences, Uppsala University Hospital, Uppsala, Sweden

**Keywords:** Kidney transplantation, Chronic active antibody-mediated rejection, Donor-specific antibody, Interleukin-6, Tocilizumab, Treatment

## Abstract

**Background:**

Chronic active antibody-mediated rejection (caAMR) in kidney transplants is associated with irreversible tissue damage and a leading cause of graft loss in the long-term. However, the treatment for caAMR remains a challenge to date. Recently, tocilizumab, a recombinant humanized monoclonal antibody directed against the human interleukin-6 (IL-6) receptor, has shown promise in the treatment of caAMR. However, it has not been systematically investigated so far underscoring the need for randomized controlled studies in this area.

**Methods:**

The INTERCEPT study is an investigator-driven randomized controlled open-label multi-center trial in kidney transplant recipients to assess the efficacy of tocilizumab in the treatment of biopsy-proven caAMR. A total of 50 recipients with biopsy-proven caAMR at least 12 months after transplantation will be randomized to receive either tocilizumab (*n* = 25) added to our standard of care (SOC) maintenance treatment or SOC alone (*n* = 25) for a period of 24 months. Patients will be followed for an additional 12 months after cessation of study medication. After the inclusion biopsies at baseline, protocol kidney graft biopsies will be performed at 12 and 24 months. The sample size calculation assumed a difference of 5 ml/year in slope of estimated glomerular filtration rate (eGFR) between the two groups for 80% power at an alpha of 0.05.

The primary endpoint is the slope of eGFR at 24 months after start of treatment. The secondary endpoints include assessment of the following at 12, 24, and 36 months: composite risk score iBox, safety, evolution and characteristics of donor-specific antibodies (DSA), graft histology, proteinuria, kidney function assessed by measured GFR (mGFR), patient- and death-censored graft survival, and patient-reported outcomes that include transplant-specific well-being, adherence to immunosuppressive medications and perceived threat of the risk of graft rejection.

**Discussion:**

No effective treatment exists for caAMR at present. Based on the hypothesis that inhibition of IL-6 receptor by tocilizumab will reduce antibody production and reduce antibody-mediated damage, our randomized trial has a potential to provide evidence for a novel treatment strategy for caAMR, therewith slowing the decline in graft function in the long-term.

**Trial registration:**

ClinicalTrials.gov NCT04561986. Registered on September 24, 2020

**Supplementary Information:**

The online version contains supplementary material available at 10.1186/s13063-024-08020-0.

## Background

A leading cause of death-censored graft loss and return to dialysis in the long-term after kidney transplantation is chronic active antibody-mediated rejection (caAMR) due to immune injury caused mainly by donor-specific antibodies (DSA) [1, 2]. Often occurring late after transplantation, caAMR is associated with chronic irreversible tissue damage and graft dysfunction [3]. It has been estimated that the cause of graft loss in the long-term is due to caAMR in as many as 50% of the cases [2].

Significant progress has been made towards an improved understanding of the molecular mechanisms of caAMR and the definition of its diagnostic criteria in recent years. It has been noted that it is the severity of renal interstitial fibrosis and not inflammation which predicts graft survival in cases of caAMR [4]. Thus, it is of great importance to manage the inflammatory process in time to avoid the development of fibrosis [3]. Three salient criteria essential for diagnosis of caAMR based on the Banff 2019 classification are (1) morphologic evidence of chronic and active lesions that include transplant glomerulopathy, severe peritubular capillary basement membrane multilayering on electron microscopy, or new arterial intimal fibrosis without another obvious cause, (2) histological evidence of antibody–endothelial interactions either by C4d deposition or at least moderate microvascular inflammation, and (3) the presence of circulating DSA, predominantly anti-HLA antibody or a DSA equivalent in the form of C4d deposits or increased expression of validated gene transcripts [5]. At least one feature from each criterion must be present for the diagnosis of caAMR. Graft histology is key to document the chronicity and extent of injury.

Despite the severity of the problem and poor outcomes for patients who develop caAMR, as well as significant advances made towards the diagnosis of caAMR, no effective therapy exists to date for this group of patients. High dose intravenous immunoglobulin (IVIG) and anti-CD20 antibody, rituximab, with or without plasma exchange, which have been used with some success for active AMR (aAMR) have also been tried for caAMR, but with unsatisfying results [6–8]. Moreover, a higher incidence of complications and adverse effects was found in the treated patients [6]. In recent small randomized controlled trials (RCTs), other therapeutic strategies such as bortezomib, a proteasome inhibitor, failed to show improvement and eculizumab, an anti-C5 antibody, indicated that it may stabilize kidney function in patients with chronic persistent DSA only during active treatment [9, 10].

The expert consensus from the Transplantation Society Working Group recommends optimizing immunosuppression and managing potential medication non-adherence in this group of patients, with the hope to control humoral activity and stabilize the graft function [3]. At our center, most patients are treated with triple therapy consisting of calcineurin inhibitor (CNI) tacrolimus + anti-proliferative agent mycophenolate acid (MPA) + prednisolone. Our usual standard of care (SOC) treatment for caAMR is optimization of these drugs. However, this treatment is insufficient in preventing graft loss in patients with caAMR, reinforcing the need for new innovative treatment strategies.

Tocilizumab (TCZ), a recombinant humanized monoclonal antibody directed against the human interleukin-6 receptor (IL-6R), has shown encouraging results in the treatment of caAMR in combination with SOC in two small uncontrolled studies [11, 12]. IL-6 is a proinflammatory cytokine that regulates inflammation as well as development, maturation and activation of T-, B- and plasma cells, and has been shown to play an important role in the pathogenesis of caAMR [13]. Moreover, a beneficial mode of action may be an altered balance between effector and regulatory T cells [14].

TCZ has been approved by the FDA and EMA for treatment of moderate to severe rheumatoid arthritis and other autoimmune diseases, with demonstrated efficacy and well-established safety profile with both subcutaneous (sc) and intravenous (iv) regimen [15–17]. However, no systematic assessment of TCZ for the treatment of caAMR has been done so far, underscoring the evidence gaps in this area and need for RCTs. Clazakizumab, another humanized monoclonal antibody that directly targets IL-6 (in contrast to TCZ that binds IL-6R), has demonstrated promise in a pilot study of caAMR [18]. Clazakizumab is not yet FDA-approved for any indication. A multi-center clinical trial investigating the efficacy of clazakizumab in treating caAMR is currently underway [19]. While both clazakizumab and TCZ inhibit IL-6/IL-6R signalling, their distinct mechanism of action may yield different effects on progression of caAMR. In our ongoing randomized study, we aim to evaluate the efficacy of adding TCZ to the SOC for the treatment of caAMR.

Apart from monitoring of kidney function, DSA and repeat biopsies, we will examine novel immunologic, histologic and molecular/genetic tools to assess responsiveness to therapy. In particular, we will evaluate the effect of study treatment on an integrative risk prediction score called iBox, a computer-based algorithm developed recently for predicting the risk of kidney graft loss and which has been validated in two large international cohorts [20].

From the transplant recipients’ perspective, failure of the kidney graft and future need of dialysis/retransplantation is a constant threat and major psychological burden. Although introducing new and stronger immunosuppression might be beneficial for the graft survival, it could have a negative impact on quality of life and adherence to medications due to potential side effects and imposed restrictions. Patient-reported outcomes (PRO) to measure the effect of a medical intervention on one or more person-centered criteria are increasingly recognized as important and should be reported in every RCT [21]. However, as per a meta-analysis of 400 RCT in kidney transplantation, PRO are reported in less than 3% of cases [22]. This can be due to the challenges of assessing the impact of research and PRO-specific issues around design, conduct, analysis and reporting [23]. Therefore, in this study, we will also assess PRO such as transplant-specific well-being, perceived threat of the risk of graft rejection and adherence to immunosuppressive medications with specific, previously developed and validated questionnaires [24–26].

To our knowledge, the INTERCEPT study is the first RCT to assess the benefit as well as the safety profile of IL-6 receptor inhibition with TCZ for the treatment of caAMR in kidney transplant recipients.

## Methods

The reporting of this protocol is based on the Standard Protocol Items: Recommendations for Interventional Trials (SPIRIT), which is found in Additional file 2 with SPIRIT checklist [27].

### Objectives

The primary objective of the INTERCEPT study is to evaluate the efficacy of addition of TCZ to SOC as compared to SOC alone in reducing the decline of graft function from baseline at 24 months after start of treatment as assessed by estimated glomerular filtration rate (eGFR) in kidney transplant recipients with caAMR.

### Design

The INTERCEPT study is a phase III investigator-driven randomized controlled open-label parallel arm multicenter study that will examine the efficacy of add-on treatment with TCZ to our SOC in comparison with the SOC alone in the treatment of caAMR. The open-label design was chosen due to the simpler logistics as well as the prohibitive expense of creating a placebo injection, especially since it is an investigator-initiated trial.

### Study setting

This in-progress investigator-initiated trial is including transplant recipients who underwent transplantation in Sweden or are currently living here and being followed up at any of the Swedish transplant centers or their affiliated regional centers. The Transplantation Center at Sahlgrenska University Hospital, Vastra Gotaland Region, is the sponsor of the study (contact: niclas.kvarnstrom@vgregion.se). The study sites include three major transplant centers at university hospitals in Gothenburg, Stockholm and Uppsala. Study drug TCZ will be prescribed by the participating sites.

### Eligibility criteria

In the study, 50 adult kidney transplant recipients who have a biopsy-proven diagnosis of caAMR according to Banff 2019 criteria [5] and are at least 12 months after transplantation will be included.

The inclusion and exclusion criteria are listed in Table 1. All biopsies with a diagnosis of caAMR performed for clinical indication will be reviewed at the main study center by two pathologists, and the patients meeting the inclusion criteria and without any exclusion criteria will be considered for the study. Repeat biopsy needs to be performed at randomization if the last biopsy is older than 12 months (+2 weeks) at randomization. In potential patients treated for caAMR with interventions like IVIG, plasma exchange, rituximab, T-cell depleting agent and/or any other medication (including another investigational drug) after the historical biopsy, a protocol transplant biopsy will be performed 2 months ± 2 weeks after treatment completion to confirm the diagnosis of ongoing caAMR. Only patients with eGFR ≥ 20 mL/min/1.73 m^2^ will be included since a greater impairment of the kidney function may mean an irreversible kidney damage. The screening eGFR determining inclusion should not be older than 1 month at the time of randomization. Infections are a concern of increased overall immunosuppression with the addition of TCZ to the SOC treatment in transplant recipients, which means that patients treated with TCZ may be at an increased risk of infections. Reactivation of viral and other serious infections (e.g. Ebstein–Barr virus (EBV) or tuberculosis (TB)) has been observed with TCZ. Therefore, to avoid the increased risk of infections, patients who are EBV-negative or with a history of TB, active/latent TB or have any active bacterial/viral infection or a history of recurring infections requiring hospitalization in the past will be excluded from the study. At screening, patients with cytomegalovirus (CMV) viremia (without any active disease) may be treated and should have at least two negative CMV-PCR values 1 week apart before they can be randomized. Similarly, patients with low-level BK-virus (BKV) viremia (≤10^3^ copies/mL or < log_10_3) at screening should have at least two negative BKV-PCR values 1 week apart before they can be randomized.

**Table 1:** Inclusion and exclusion criteria

Inclusion Criteria	Exclusion Criteria
1. Written informed consent 2. Recipient of living donor or deceased donor kidney transplant 3. Age ≥18 4. At least 12 months post-transplant at randomization 5. Biopsy-proven diagnosis of caAMR (not older than 1 year at randomization) 6. eGFR ≥20 ml/min/1.73 m^2^ (not older than 1 month at randomization) 7. Epstein-Barr Virus (EBV) IgG-positive 8. Female participants of childbearing potential: use of adequate contraception and a negative pregnancy test 9. Subjects with previous COVID-19 infection must meet the following conditions: - Asymptomatic > 1 month before the start of screening - Re-established immunosuppressants > 2 weeks prior to the start of screening	1. Recipient of multi-organ transplants 2. eGFR <20 ml/min/1.73 m^2^ 3*. De novo* or recurrent renal disease, if it is considered to be the predominant cause of current graft dysfunction 4. Active viral infections^a^ 5. Ongoing serious infections 6. History of recurrent infections requiring hospitalization 7. Signs of post-transplant lymphoproliferative disorder 8. History of tuberculosis, active or latent disease 9. Abnormal liver function tests (ALT, AST or bilirubin > 1.5 x upper limit of normal) 10. Other significant liver disease 11. Neutropenia (<2 x10^9^/L) or thrombocytopenia (<100 x10^9^/L) 12. Signs of malignancy. Exceptions are basal or squamous cell carcinoma or non-malignant melanoma 13. History of malignancy, unless subject has been considered to have fully recovered from malignancy since > 2 years, without any signs of relapse 14. History of diverticulitis, diverticulosis, gastrointestinal perforation, or inflammatory bowel disease 15. Substance abuse 16. Serious medical or psychiatric illness 17. Mental inability, reluctance, or difficulties in understanding the informed consent 18. Woman of childbearing potential not applying inclusion criteria number 8 19. Current or recent (within last 3 months) participation in another clinical drug trial

Details are provided under Eligibility criteria in Methods section

*ALT* Alanine transaminase, *AST* Aspartate transaminase, *caAMR* Chronic active antibody-mediated rejection, *eGFR* Estimated glomerular filtration rate

^a^Such as BK virus (BKV), cytomegalovirus (CMV), SARS COV-2 (COVID-19), EBV, hepatitis C virus (HCV) or hepatitis B virus (HBV) infections, based on polymerase chain reaction (PCR) testing

Moreover, due to already known side effect of TCZ such as leukopenia, thrombocytopenia and hepatic dysfunction, patients who exhibit abnormal liver function tests (alanine transaminase (ALT), aspartate transaminase (AST), bilirubin > 1.5 × upper limit of normal), other significant liver disease as per investigator’s opinion, neutropenia (<2 × 10^9^/L) or thrombocytopenia (<100 × 10^9^/L) will be excluded. Woman of childbearing potential who is unwilling/unable to use an adequate and safe method, e.g. hormonal therapy, to avoid pregnancy for the entire study period and for up to 8 weeks after the last dose of study drug will also be excluded.

### Screening

Screening will take place within 6 months before the randomization. All relevant information will be obtained from their electronic medical charts as well as databases of the respective transplantation centers, pathology and clinical histocompatibility laboratories. Physical examination, chest X-ray, study-related blood samples (including mGFR using iohexol clearance test) as well as a urine sample will be taken, and each subject’s eligibility will be established by a physician at the respective study center before inclusion and randomization of a patient to treatment arms. In patients with an eGFR between 20 and 25 ml/min/1.73 m^2^ or experiencing a rapid decline in kidney function, eGFR will be repeated at the discretion of the treating physician. In patients who are deemed ineligible for participation at screening, rescreening will be considered on an individual basis and must first be approved by the sponsor.

### Randomization

Eligible participants, after they have provided informed consent, will be consecutively randomized at the point of randomization by the study coordinator at each site. This will be achieved using a computer program embedded in the electronic case report form (eCRF), assigning participants to either of the two study arms in a 1:1 ratio and stratified by study site. The trial is non-blinded. If a subject discontinues their study participation, their subject code will not be reused, and the subject will not be allowed to re-enter the study again. Randomization will be to one of two treatment groups as follows: Arm A (SOC + TCZ): SOC (as below) + TCZ (162 mg every week, SC administration). Arm B (SOC): tacrolimus (target concentration 6 ±1 μg/L) + MPA (1–2 g/day as tolerated) + steroids (prednisolone not less than 5 mg/day), all oral administration.

The patients who do not tolerate either tacrolimus and/or MPA in the SOC treatment in either arm may be treated with other immunosuppressive drugs. The following regimen will be considered equivalent:

- Tacrolimus can be replaced with another calcineurin inhibitor, cyclosporine, where a target concentration of 80–120 ng/ml.
- MPA can be replaced with another antimetabolite, azathioprine (optimally tolerated dose based on leukocyte count) or an mTOR inhibitor, everolimus, with a target concentration of 3–4 μg/l.

**Fig. 1:**
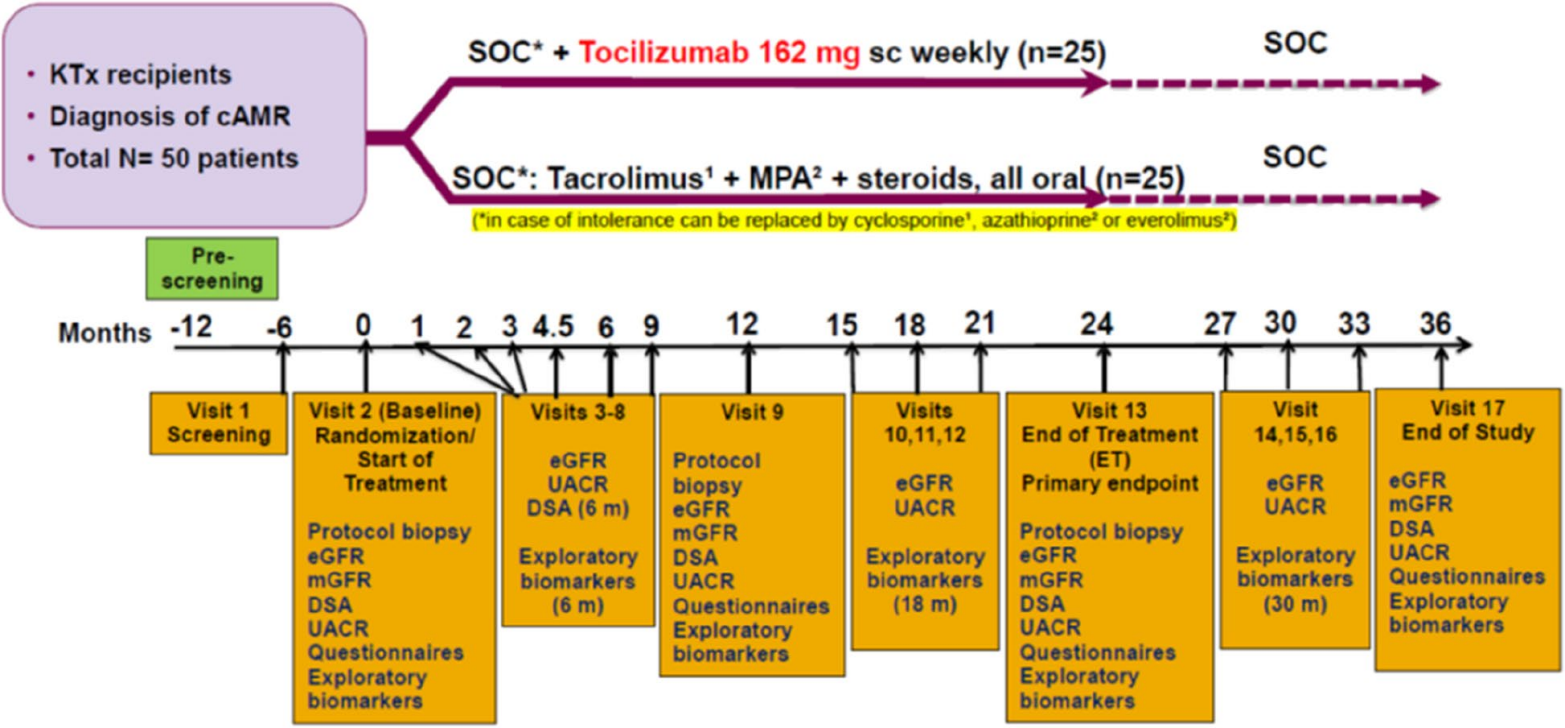
Flow chart of the Intercept-study. DSA donor-specific antibodies, eGFR estimated glomerular filtration rate, KTx kidney transplant, m months, mGFR measured glomerular filtration rate, MPA mycophenolic acid, sc subcutaneous, SOC standard of care, UACR urine albumin:creatinine ratio

The study treatment will be continued for 24 months after which both groups will continue only with the SOC. All patients will be followed up for an additional 12 months. A study design flowchart is shown in Fig. 1 and a detailed intervention plan can be found in Table 2, the SPIRIT figure. Enrolment will be continued until the required sample size is achieved.

**Table 2:**
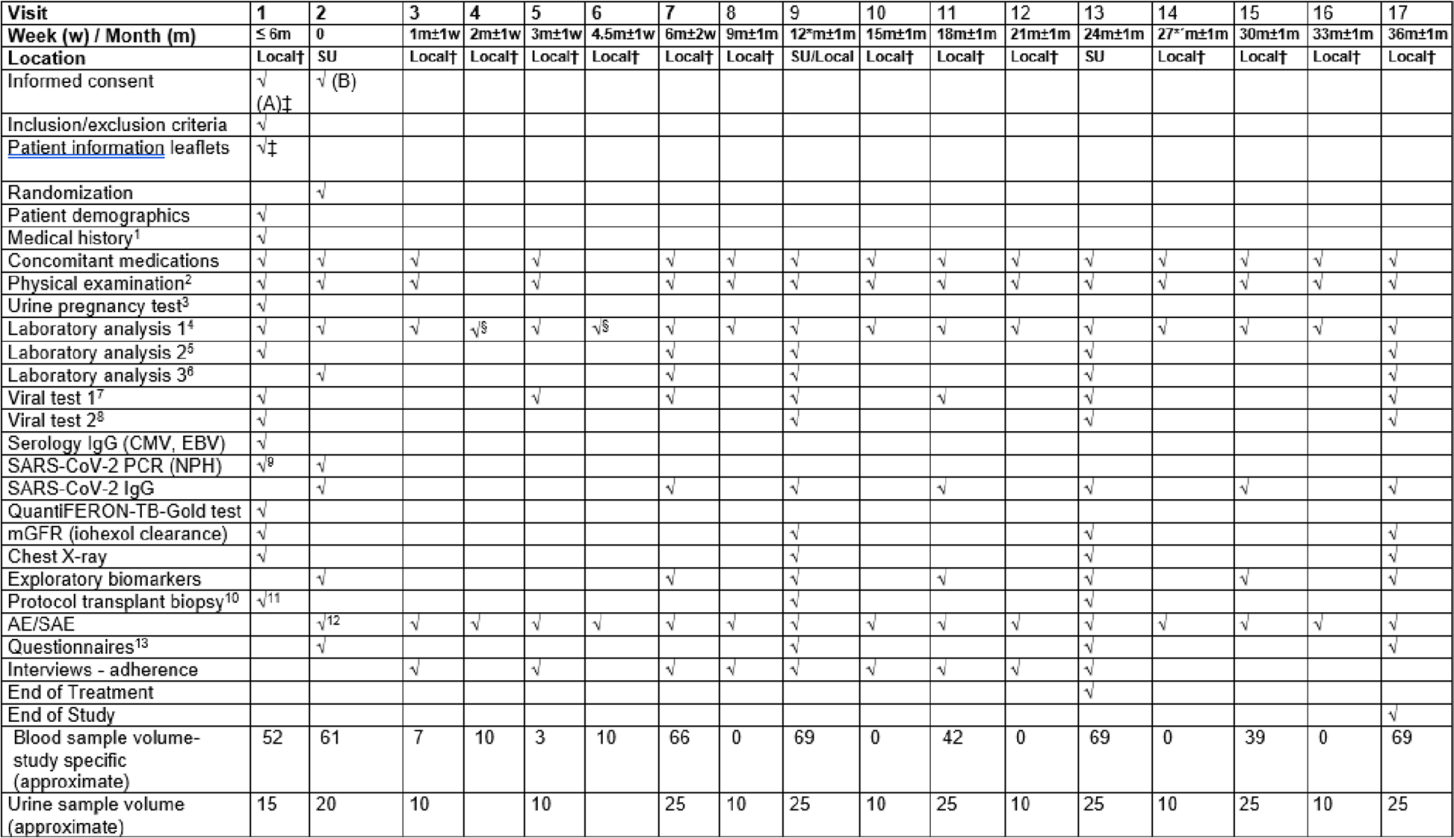
The SPIRIT figure

*AE* Adverse events, *CM*V Cytomegalovirus, *EBV* Ebstein–Barr virus, *m* month, *PCR* Polymerase chain reaction, *SAE* Serious adverse events, *w* week

† Local = regional outpatient clinic or at SU

‡Done prior to visit 1, as part of pre-screening

§Without urine

^1^Including also collection of information about baseline pretransplant characteristics, transplant-related characteristics and review of previous biopsies

^2^Physical examination by a physician that includes weight, height, body mass index, vital signs

^3^Only for women of childbearing potential (urine)

^4^Laboratory analysis 1: haemoglobin, complete and differential white blood counts, platelet count, glucose, creatinine (eGFR), urea, sodium, potassium, liver transaminases, bilirubin, alkaline phosphatase, fibrinogen, tacrolimus concentration, C-reactive protein, urine dipstick, UACR;

^5^Laboratory analysis 2: serum electrophoresis

^6^Laboratory analysis 3: fasting lipid profile, DSA, T-lymphocytes cell count and subsets (CD4, CD8, memory and naive CD4/CD8, T-regs CD4), B-lymphocyte cell count, peripheral blood mononuclear cells (PBMCs), ABO-antibody titer (in ABO-incompatible transplants), total IgG, tetanus IgG

^7^Viral test 1: CMV, BKV, by PCR

^8^Viral test 2: HBV, HCV, EBV by PCR, HBsAg

^9^Only if patient has symptoms suggestive of COVID-19

^10^APTT and INR will be done before the biopsy (2.7 ml blood required)

^11^Only for those whose last biopsy will be older than 12 months at randomization

^12^From protocol biopsy if done during visit 1

^13^Organ Transplant Symptoms and Well-being Instrument (OTSWI), Basel Assessment of Adherence with Immunosuppressive medication Scales (BAASIS©), Perceived Threat of the risk of Graft Rejection (PTGR)

### Sample size

The sample size calculation is based on our preliminary analysis of caAMR patients at the SU and assumption of an initial mean eGFR of 48 ± 15 ml/min, with a mean decline (slope) of −7.5 ml/year, a standard deviation of 15 throughout the visits and an intra-patient correlation of 0.85. To uncover a difference of 5 ml/year in the eGFR slope in the two arms, 25 patients would be required in each arm including 10% dropouts, using 1000 simulations, with a power of 80% at a significance level alpha of 0.05, based on eGFR measurements every 3 months using the above specified repeated measures linear model. The sample size is based on decline in eGFR only and therefore, no corrections for multiple comparisons are required.

### Feasibility of patient recruitment

The study’s patient population encompasses all kidney transplant recipients in Sweden, involving all four transplant centers with approximately 6000 active recipients and around 450 kidney transplants conducted annually [28]. Therefore, this sample size is considered achievable due to expected throughput of eligible participants from all centers. In addition, we have implemented various surveillance strategies and new clinical routines for active screening of DSA and performance of early biopsies in transplant patients to facilitate early diagnosis and identification of potential candidates.

### Intervention

TCZ as a SC regimen will be self-administered by the patients on the same weekday and at the same time. Prefilled injection pens of TCZ will be prescribed electronically to the patients by the study centers. Guidelines for at-home administration of SC injections of TCZ will be implemented in this study in accordance with the routine clinical practice in patients with rheumatoid arthritis. These include training of patients in SC TCZ administration for the first injection by the study coordinators. Adherence to the treatment will be evaluated in congruence with each study visit by patient interviews, assessment of the patient diaries and counting of returned used injection pens.

Close follow-up of all the study subjects will be performed to control or mitigate potential risks. Subjects shall be closely monitored clinically after start of intervention (baseline) at each clinical visit. Physical examinations will be performed, and blood and urine samples will be taken regularly and evaluated. In order to promote participant retention and complete follow-up, the study coordinators will have close contact with the patients via phone and shall send written reminders for every visit and annual protocol biopsies to patients in both arms as well as their regional outpatient clinics. The sponsor will closely monitor recruitment rates and ensure data completeness, through a combination of on-site visits and central monitoring at study sites.

### Prohibited/permitted interventions

Since there is no established treatment for caAMR, the patients in the SOC arm will not receive any added treatment. The following treatments are prohibited for the duration of the study in either arm for the treatment of caAMR: any other anti-IL-6/IL-6R monoclonal antibodies, eculizumab, proteasome inhibitors, IVIG (except for treatment of hypogammaglobulinemia or BKV infection during the study) or plasma exchange. For acute T cell-mediated rejection, intravenous methyl prednisolone or T-cell-depleting agents (in case of steroid resistance) may be used for treatment during the study.

### Risk mitigation

If a bacterial infection is suspected in any patient, in addition to the routine markers of infections such as white cell counts and CRP, levels of procalcitonin, a marker of infection released by alternate pathways, which should not be affected by IL-6 inhibition, will also be checked in blood [29]. Specific dosage modifications will be made or the drug discontinued in case of adverse events such as cytopenia and elevated liver enzymes.

Furthermore, patients will be made aware of the symptoms potentially indicative of diverticular disease and instructed to alert their healthcare provider as soon as possible if these symptoms arise due to the acknowledged side effect of gastrointestinal perforation. Should this develop, TCZ will be discontinued. Risk factors for cardiovascular disease (e.g. hypertension, hyperlipidemia) will be managed as part of their routine care. Due to interactions of TCZ with statins and calcium channel blockers, patients on these drugs might need dose adjustments. Dose modifications will also be done for neutropenia, thrombocytopenia, elevation of liver enzymes or hypofibrinogenemia which are common side-effects of TCZ. If eGFR falls below 20 ml/min, the decision to continue tocilizumab (TCZ) should be at investigator’s discretion, focusing on participant safety. Significant eGFR reduction may prompt a biopsy for identifying graft dysfunction causes. The study drug treatment will be discontinued prematurely if the patients experience any of the following: pregnancy, diverticulitis, gastrointestinal perforations, persistent neutropenia < 0.5 cells × 109/L, thrombocytopenia < 50 cells × 109/L, elevated liver enzymes ALT or AST > 3 to 5 × ULN or low fibrinogen levels < 50% of the lower limits of normal or malignancies (except local BCC or SCC of the skin or carcinoma in situ of the cervix uteri that have been excised and cured). Study drug treatment may also be discontinued prematurely at the investigator’s discretion due to, e.g. other adverse events suspected to be related to TCZ or protocol violation. Clear guidelines for dose modifications or discontinuation of TCZ are provided in the study protocol. Patients discontinuing study treatment prematurely will continue participating in the study, attending visits and following protocols. They will be allocated in the intent-to-treat (ITT) group. Study termination criteria include loss to follow-up (e.g. due to relocation) or complete withdrawal of consent for further data collection from medical charts.

### Outcome parameters

#### Primary endpoint

The primary endpoint is the change in slope of estimated glomerular filtration rate (eGFR) at 24 months after start of treatment using MDRD formula [30] without adjusting for the race component in kidney transplant recipients with caAMR.

#### Secondary endpoints

The secondary endpoints are specified in Table 3.

**Table 3:** Study endpoints

**Primary endpoint:** Mean rate of change in eGFR (eGFR slope) from baseline to 24 months after start of treatment	
**Secondary endpoints:** 1. Change from baseline in mean iBox risk prediction score at 12 and 24 months 2. Safety: incidence, nature and severity of adverse events (AE) during 24 months of treatment period 3. Evolution of DSA (MFI) at baseline, 12, 24 and 36 months 4. Histologic changes in biopsy at 12 and 24 months 5. Changes in proteinuria (UACR) at 12, 24 and 36 months 6. Changes in renal function at 12, 24 and 36 months, assessed by mGFR using iohexol clearance 7. Changes in renal function at 12 and 36 months, assessed by eGFR 8. Incidence of patient survival at 12, 24 and 36 months 9. Incidence of death-censored graft survival 12, 24 and 36 months 10. Possible changes of experienced transplant-specific well-being, symptom burden, perceived threat of the risk of graft rejection and adherence to immunosuppressive medications at 12, 24 and 36 months after start of treatment	

*DSA* Donor-specific antibodies, *eGFR* Estimated glomerular filtration rate, *MFI* Mean fluorescence intensity, *UACR* Urine albumin:creatinine ratio

##### iBOX

Change from baseline in the mean composite iBox score will be determined at 12 and 24 months after start of treatment. The full iBox risk score is based on the following eight clinical/histological/immunological risk factors: time from transplant to evaluation, eGFR, proteinuria, four histological parameters based on Banff scoring (interstitial fibrosis/tubular atrophy, microcirculation inflammation, interstitial inflammation and tubulitis, transplant glomerulopathy) and iDSA MFI category (Table 4) [20]. The iBox score will be generated using a computerized algorithm and the mean score will be assessed as a continuous variable. An improvement/stabilization of score will be considered as response to treatment and will also be assessed as a yes/no categorical variable. A simplified iBox score based only on four functional and immunological data will also be assessed.

**Table 4:**
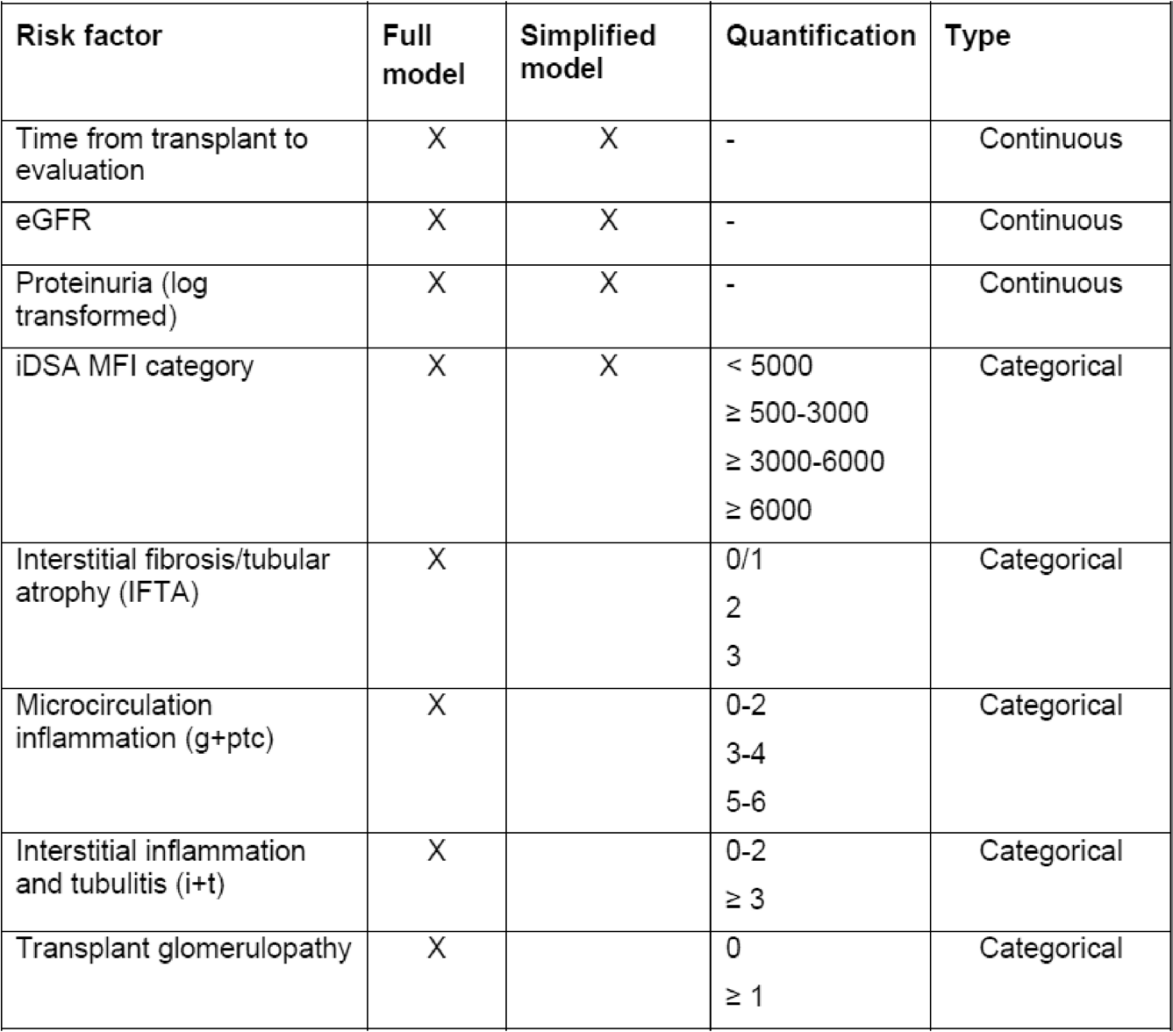
iBox variables

*DSA* Donor-specific antibodies, *eGFR* Estimated glomerular filtration rate, *MFI* Mean fluorescence intensity

##### Evolution of DSA

The testing for DSA will be performed in the tissue typing lab at the SU using whole blood samples. Commercially available single antigen flow-bead (SAB) testing One Lambda, Canoga Park, CA (USA), will be used to test for donor specificity, mean fluorescence intensity (MFI), HLA-class and complement C1q-binding capability. DSA MFI is measured as a continuous variable and considered positive if MFI is > 1000. If several DSAs are present, the cumulative MFI (cMFI) will be evaluated by adding together the MFI of each single antibody, as well as the MFI of immunodominant DSA (iDSA), which are defined as the strongest DSAs (the ones with highest MFI) detected in the patients’ sera. Response will be defined as reduction/stabilization in cMFI and/or iDSA at 24 months compared to baseline and assessed as a categorical variable.

##### Protocol transplant biopsies

After the inclusion biopsy at baseline, ultrasound-guided percutaneous protocol biopsies will be performed at 12 and 24 months, after exclusion of a coagulation disorder or thrombocyte count below 80% of the normal value. Three cores of biopsies will be taken with a 16 gauge. All biopsies will be sent to the respective pathology laboratories for further processing and evaluation. The pathologists are blinded regarding which arm the patients are in. Biopsy evaluation will be made through standard paraffin-embedded sections including immunohistochemical complement C4d staining, using a monoclonal antibody (BioSite, rabbit monoclonal antibody, clone A24-T). A diagnosis of caAMR will be made if there is evidence of being both chronic and active, presumably antibody-mediated tissue damage (Table 5), as defined by the Banff 2019 criteria [5] and the biopsies will be scored accordingly.

**Table 5:**
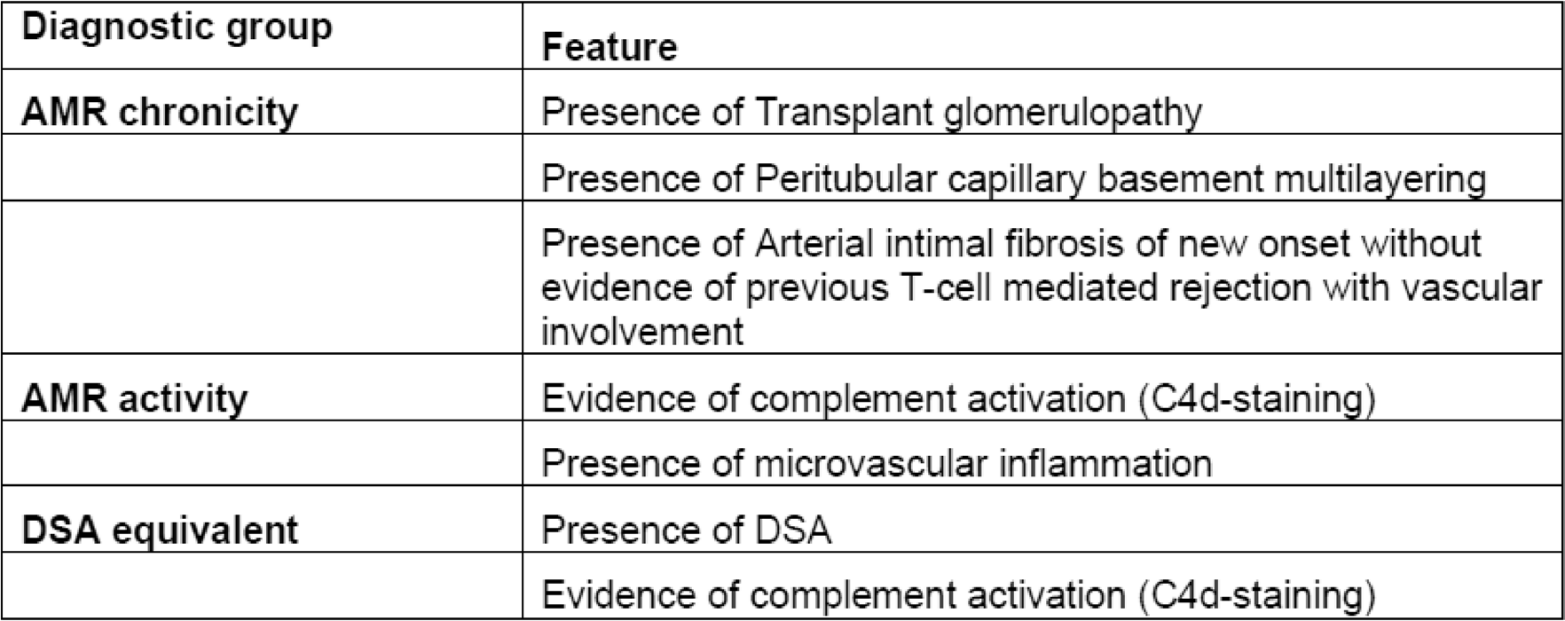
Diagnostic criteria of caAMR^a^

*AMR* Antibody-mediated rejection, *DSA* Donor-specific antibodies, *caAMR* chronic active antibody-mediated rejection

^a^At least one feature from each of the three criteria must be present for the diagnosis of caAMR

All biopsies which show aAMR without evidence of chronic changes by light microscopy will be assessed by electron microscopy in order to exclude or confirm presence of peritubular capillary basement membrane multilayering or glomerular basement membrane double contours. If the criteria for caAMR are no longer fulfilled in the follow-up biopsies, response to therapy is assumed. The response will be assessed as a yes/no categorical variable. In all biopsies, which still meet the required criteria for caAMR, means of individual Banff lesion scores will be compared between the baseline biopsy and the 12- and 24-month biopsies as continuous variables.

##### Questionnaires

We also aim to assess in this study the three PRO mentioned above. We will also evaluate the differences in these PRO in relation to age, sex, occupation, civil status, educational status and type of treatment arm, and fear of graft rejection in relation to biopsy or type of treatment arm at baseline and at 12, 24 and 36 months. The following validated qualitative research instruments will be self-reported by the patients, either directly online or on paper, at baseline and every 12 months until 36 months. Permissions to use these instruments in this study have been obtained.

- The OTSWI (Organ Transplant Symptoms and Well-being Instrument)

The OTSWI is a multi-item questionnaire that has been developed to measure symptom prevalence, symptom distress and transplant-specific well-being after organ transplantation. It will be used to assess transplant-specific symptoms and well-being during the whole study period [24].

b) The BAASIS© (Basel Assessment of Adherence with Immunosuppressive medication Scales) is a self-report instrument for assessingherence to immunosuppressive drugs. Possible associations between non-adherence and rejection and/or development of DSA will be explored [26].
c) The PTGR (Perceived Threat of the risk of Graft Rejection) is a multi-item questionnaire that measures the phenomenon labelled, ‘graft-related threat’ (GRT), ‘intrusive anxiety’ (IA) and ‘lack of control’ (LOC). Possible associations between the three different PTGR parts and OTSWI and rejection will be explored [25].

### Statistical analysis

The primary analysis of eGFR decline by therapy will be performed using a repeated measures linear model (two sided at alpha 5%) adjusting for eGFR at baseline and donor status (living versus deceased) as fixed effects and center as random effect. Visits/time will be included in the model as a linear variable and the *P*-value of the interaction term of decline in eGFR and visit/time will be the primary analysis. The estimated treatment difference with the associated 95% confidence interval (CI) and *P*-value will be presented. Two-sided *P*-value less than 0.05 will be considered significant.

The hierarchical testing procedure below is introduced to guarantee that the probability of Type 1 error is <5% for all confirmative statements. The order of the hierarchical testing procedure will be as follows:

1. eGFR decline (primary efficacy analysis)
2. Change from baseline in mean composite iBox risk prediction score at 24 months

If the first analysis is significant, the probability mass 0.05 will go to the second analysis. If the first analysis is non-significant, no analysis will be confirmative.

Results from the other endpoints and from subgroups shall be considered hypothesis generating only. *P*-values for the endpoints other than eGFR (primary) and iBox, if presented, should be interpreted in a descriptive fashion only and cannot be considered as significant.

Secondary endpoints will be analysed in an exploratory manner, using appropriate parametric and non-parametric statistical methods. For comparisons between two groups Fisher’s exact test will be used for binary response data, Mantel–Haenszel chi square test for ordered categorical data, the *t*-test for independent samples or Mann–Whitney *U* test for continuous data. Continuous data will be expressed using mean (standard deviation) or median (interquartile range) and categorical data as numbers (frequencies); 95% confidence intervals will be calculated when appropriate. Missing data will be analysed with regard to reasons and pattern, and sensitivity analyses will be performed based on various assumptions regarding the pattern. The results from the primary and secondary endpoints will also be stratified and presented according to sex.

The ITT population will consist of all randomized patients who take at least one dose of assigned treatment and have at least one follow-up with measurements. The analysis of all efficacy data will be performed on the ITT population. The per-protocol (PP) population will include all ITT patients without any major protocol deviations, who did not have to stop the study drug for >4 weeks due to side-effects, and who had ≥80% compliance with study drug (with dose adjustments if required) while on treatment (up to discontinuation for patients whose treatment is terminated early). The PP analysis will be used to assess the robustness of the ITT analysis results of primary and secondary efficacy data.

The statistical analysis will be performed using the commercially available software SAS v9.4. A detailed statistical analysis plan will be written where all populations, variables, and statistical methods will be described. The total number of subjects per arm (*n* = 25) considers a total dropout rate of approximately 10%. These patients will still be included in the final ITT analysis. For the patients who drop out, their data will be analysed up until the date of last available clinical data.

### Data Monitoring and Safety Board

To secure the safety of the INTERCEPT study population and integrity of the study, the totality of data will be reviewed on a regular basis by an independent Data Monitoring and Safety Board (DMSB). The DMSB will consist of two physicians and one statistician, neither with any other involvement in the study. The first formal interim analysis meeting will be held to review data relating to treatment efficacy, patient safety and quality of trial conduct when approximately 40% of the patients (~20 subjects) have reached the time-point for the primary endpoint assessment and the data are cleaned. Subsequently, meetings will be held after the inclusion of all patients is complete, upon the attainment of the primary endpoint in all enrolled patients and in the event of any safety concerns. The DMSB will determine if amendments to the protocol or changes in study conduct are required and may consider terminating the trial if there are major safety concerns.

### Quality control and assurance

The study will undergo thorough monitoring by an independent study monitor before, during and after the study to ensure protocol adherence and proper collection, documentation and reporting of that data and all essential documents. This will be conducted in accordance with International Council for Harmonisation (ICH)-GCP E6 (R2) standards and applicable ethical and regulatory requirements. The investigator will provide access to all source documents, including eCRFs and other protocol-related materials. Patient confidentiality will be maintained per local regulations. The monitor will regularly review eCRFs based on a defined monitoring plan to verify adherence to and completeness of protocol as well as the validity and accuracy of entered data. Throughout the study, the monitor will conduct predefined visits at the study center, verifying informed consent, adherence to inclusion/exclusion criteria, documentation of severe adverse events and the recording of the main efficacy, safety and tolerability endpoints.

#### Data management

All data collection in the study will be through an eCRF. Investigators are responsible for accurate data registration and corrections per the study protocol. The independent external monitor will randomly verify data accuracy by comparing it with source documents. This verification ensures compliance with the International Council for Harmonisation (ICH)-GCP E6 (R2) and relevant guidelines and ethical regulations. If no inconsistencies are found, the appropriate eCRFs are collected. The investigator will sign the completed eCRF, and a copy of it will be archived at the study sites. Queries or responses will be processed into the database by the Data Management team.

Investigators are responsible for ensuring that all data in the eCRFs and Data Clarification Forms are accurate, complete and legible, and that all entries are verifiable with source documents. Source documents for each subject in the study will be retained, and a document defining classified source data will be included in the Investigator Site File (ISF) at each site. Data management will be overseen by the sponsor representative/PI. The study database will be soft-locked upon receipt and cleaning of all specified data in the study protocol. It will be hard-locked after a (blind) data review meeting, where all data-related decisions have been made and reflected in the database.

### Ethical considerations

The study will be executed in alignment with the study protocol, ICH-GCP E6 (R2), the latest DMSB ensure the safety and integrity of the study subjects as well as the quality of the data collected. Patients who are willing to participate will be given adequate oral and written information about the study, its purpose, any risks and benefits as well as inclusion and exclusion criteria, as well as the informed consent form (ICF) by licensed physicians at the study center. In case any new ancillary studies are planned with the already stored biological samples, a new informed consent will be obtained. Each subject who participates in the study will be identified by a subject number on a subject identification list. Any amendments that occur throughout the study will be passed on to affected parties.

Patient data and samples for this study will be handled with confidentiality measures and GDPR compliance. Each study participant will be assigned a subject number for identification, and blood and urine samples collected for future biomarker research will be coded with a unique study identification number. The identification/code list will be securely stored in locked cupboards at the Clinical Trial Units of the respective Transplantation Centers to prevent unauthorized access. Only study personnel will have access to the code list. Samples collected from study patients will adhere to the Biobank Act and be registered with Biobank West (www.biobankvast.se), under Region Vastra Gotaland. The samples will be assigned a unique QR code in the biobank, linked electronically to personal numbers and stored pseudonymized to protect participant identification. Access to the code key and samples will be restricted to study personnel only. Samples will be stored for at least 10 years post-study, used for study-related purposes, and then destroyed. Identification lists will be kept securely for the same duration and destroyed afterward.

All data, including informed consent, completed eCRF, protocol and final report, will be encrypted for accurate reporting and stored at the study center for at least 10 years post-trial, as per Swedish law. Information processed by the sponsor will be pseudonymized with a study identification number, ensuring anonymity in the presentation or publication of study results.

The investigators intend to communicate the trial results to participants, healthcare professionals and the public via a summarized manuscript published in a scientific journal. All subjects are insured through the Swedish patient insurance and will receive post-trial care if they suffer any harm from trial participation. Paid sick leave is possible if deemed necessary.

### Study registration

The final study protocol including the final versions of the informed consent form and other information provided to subjects for the INTERCEPT study have been approved by The Swedish Ethical Review Authority (Etikprövningsmyndigheten, Dnr 2020-03156). It has also been approved by the Medical Products Agency (Läkemedelsverket, Dnr 2019-004302-10). The study has been registered in a public clinical trial database, ClincalTrials.gov **(**NCT04561986).

## Discussion

In kidney transplantation, caAMR represents a big challenge to the existing immunosuppressive strategies. Due to the lack of established effective treatment for caAMR to date, we are initiating this RCT to study the potential efficacy of the promising novel treatment with TCZ.

Until now, high dose IVIG and anti-CD20 antibody, rituximab, with or without plasma exchange has been used by many centers as rescue therapy for caAMR, although evidence of the efficacy of this regimen is not strong [6]. IVIG/rituximab regimen has shown stabilization of caAMR and reduction in DSA in a few non-controlled studies [31]. However, the neutral results of a multicenter RCT from Spain that included 25 patients argue against a relevant therapeutic effect of such a regimen [8]. Nonetheless, these results should be interpreted with caution since the study was underpowered and the lack of response due to selection of a population with too advanced histological damage cannot be discarded. Supporting these findings, improvement in graft survival was not observed when comparing 39 untreated with 23 patients treated with IVIG/rituximab in a recent study. There was even a higher incidence of complications and adverse effects in the treated patients [6]. An observational study of 123 patients where different strategies combining steroid boluses, IVIG, rituximab, plasma exchange and thymoglobulin as a treatment approach showed no improved outcome of caAMR in kidney transplant recipients [32]. A systematic review which evaluated seven studies with caAMR concluded that there was no evidence for benefit with rituximab [33].

A few other novel treatments for caAMR have been tested in recent years. Eculizumab, a humanized monoclonal (IgG2/4κ) antibody, has been evaluated in a pilot RCT (15 patients; 10 treatment arm, 5 control arm) with caAMR where eculizumab was given for 6 months. However, the eculizumab treatment stabilized kidney function only during active treatment [10], the study population was very heterogeneous with large variations in the baseline kidney function, as well as the medication is very expensive. In another RCT that investigated bortezomib, a proteasome inhibitor, in 44 patients (21 treatment, 23 placebo) with caAMR, bortezomib treatment failed to prevent GFR loss, improve histologic features or reduction of DSA [9]. The two study arms had a similar incidence and spectrum of infectious complications. However, bortezomib was associated with gastrointestinal toxicity as well as bone marrow suppression.

Interestingly, TCZ, an anti-IL-6 receptor antibody, has shown promise in two uncontrolled observational studies for the treatment of caAMR. In the first pilot study, 36 kidney transplant recipients with caAMR were treated with iv TCZ. The authors reported an acceptable safety profile, improved graft and patient survival, stabilization of kidney function, a significant reduction in DSA levels over time and a decrease in microcirculation inflammation in follow-up biopsies 1 year after tocilizumab treatment [11]. Another recent study of 15 patients with caAMR treated with TCZ as a first-line therapy showed that despite advanced transplant glomerulopathy (TG) in most patients, eGFR and proteinuria stabilized during the median follow-up of 20.7 months, with a significant reduction in DSA. Protocol biopsies after 6 months demonstrated significant amelioration of microvascular inflammation and no progression of TG, C4d deposition or interstitial fibrosis (IF)/tubular atrophy (TA) [12]. However, no systematic assessment has been done so far underscoring the evidence gaps in this area and need for RCTs.

In the INTERCEPT study, patients will be randomly assigned to TCZ + SOC or SOC treatment for 24 months, followed by 12 months of monitoring. We have chosen the change in eGFR (eGFR slope) as the primary endpoint, as a clinical trial designed to show a difference in slope of GFR decline between randomized treatment arms could require a smaller sample size and shorter follow-up than a trial designed to show a difference in the occurrence of hard clinical endpoints such as graft loss or mortality. Difference in eGFR slope has been associated with increased risk of kidney failure and has been confirmed as a valid endpoint in chronic kidney studies [23, 34]. In kidney transplant recipients, reduction in the rate of change in eGFR post-diagnosis of caAMR has been shown to improve death-censored graft survival [35]. This outcome measure may circumvent the need for lengthy follow-up and/or recruitment of very large numbers of patients. We estimate that at least a 2-year study duration is required to notice a change in the kidney function in patients with caAMR. Patients with eGFR < 20 mL/min/1.73 m^2^ will not be included since a greater impairment of the kidney function may mean an irreversible kidney damage.

Identifying novel more sensitive diagnostic and prognostic precision medicine tools are essential requirements for assessing the graft injury and improve our ability to monitor patients, predict graft failure and responsiveness to therapy. Hence, as secondary endpoints, we will utilize the prediction score iBox to improve risk stratification for transplant outcomes and improve the management of patients with caAMR. The iBox score is emerging as a surrogate endpoint for the development of new clinical studies in kidney transplantation as it should be able to significantly reduce the time, and therefore the cost, of clinical studies by providing an early-stage reliable prediction of the long-term graft survival. Thus, the iBox score will allow for comparative efficacy assessments between the control and intervention arms in our study and guide decision-making regarding the long-term efficacy of the new therapeutic intervention [20, 36]. By combining functional, histological and immunological parameters, the tool generates probabilities of graft loss up to 10 years after patient evaluation. Combining many factors into a well-validated model provides broader biological insights, better reflect the complexity of late graft failure and are likely to predict long-term outcome than using individual components in isolation.

By examining PRO such as patients’ adherence, perceived risk of graft rejection and their well-being in this study, we hope to find out more about the complexity and demands that one experiences in terms of participating in such trials with new interventions. There is little data assessing sex-related differences in psychological well-being and adherence to treatment in patients treated for caAMR. Furthermore, sex-related differences in responsiveness to treatment of caAMR are not known despite women having a higher prevalence of anti-HLA than men [37] and being at increased risk for caAMR [38]. Therefore, we also plan to analyse sex-related differences in these aspects in the current study while also considering the patients’ current civil, educational and occupational status.

Safety of a novel treatment is always of concern in clinical trials and therefore it is important to undertake risk mitigation strategies during the study. Patients treated with TCZ may be at an increased risk of infections because of overall increased immunosuppression. Yet, from another perspective, underimmunosuppression is a known contributing factor to caAMR and supports the idea of increased immunosuppressive treatment. Cytopenia and elevated liver enzymes are known adverse advents of TCZ for which specific dosage adjustment or discontinuation of the drug will be done. Diverticulitis and gastrointestinal perforation are rare side-effects of treatment with TCZ. Therefore, as in previous studies with TCZ, patients with any history of diverticulitis or inflammatory bowel disease will be excluded in our study. Furthermore, patients will be made aware of the symptoms potentially indicative of diverticular disease and instructed to alert their healthcare provider as soon as possible if these symptoms arise. In order to reduce the risk of adverse events, an elaborate schedule of visits and tests as well as mitigation strategies have been included in the study protocol. The totality of data will be reviewed on a regular basis by the DMSB.

To the best of our knowledge, the INTERCEPT study is the first controlled prospective RCT for the treatment caAMR with IL-6 inhibitor TCZ in kidney transplant recipients. Therefore, if the results of our study show favourable graft function with TCZ, it will have major clinical implications by identifying a novel effective therapy for treatment of these patients who would otherwise lose their graft. Thus, the planned study can improve graft survival, reduce patient morbidity and mortality, improve our understanding of patient-related outcomes and their quality of life. Furthermore, identification of novel tools will allow better assessment of graft injury and risk stratification for graft failure in caAMR leading to improved individualized management.

### Trial status

Protocol version number: 1.5

Date: February 13, 2023

Start of recruitment: February 2022

End of recruitment: December 2024

### Supplementary Information

**Additional file 1.** Statistical analysis.

**Additional file 2.** SPIRIT checklist.

## Data Availability

All current and available data can be found at ClinicalTrials.gov ID: NCT03459287.

## Abbreviations

AE: Adverse event = undesired medical event
AMR: Antibody-mediated rejection
BAASIS: Basel assessment of adherence with immunosuppressive medication scales
BKV: BK virus
caAMR: Chronic active antibody-mediated rejection
CI: Confidence interval
cMFI: Cumulative mean fluorescence intensity
CMV: Cytomegalovirus
CNI: Calcineurin inhibitor
COVID-19: Coronavirus disease of 2019
CRF: Case report form
DMSB: Data monitoring and safety board
DSA: Donor-specific anti-HLA antibodies
EBV: Epstein–barr virus
eCRF: Electronic case report form
eGFR: Estimated glomerular filtration rate
EMA: European medical agency
FDA: Food and drug administration
GCP: Good clinical practice
GDPR: General data protection regulation
GRT: Graft-related threat
HBV: Hepatitis B virus
HCV: Hepatitis C virus
HLA: Human leukocyte antigen
IA: Intrusive anxiety
ICH: International council for harmonisation
IgG: Immunoglobulin G
iDSA: Immunodominant donor-specific anti-HLA antibodies
IL-6: Interleukin-6
ITT: Intention-to-treat
iv: Intravenous
IVIG: Intravenous immunoglobulin
MFI: Mean fluorescence intensity
mGFR: Measured glomerular filtration rate
MMF: Mycophenolate mofetil
MPA: Mycophenolic acid
OTSWI: Organ transplant symptoms and well-being instrument
PCR: Polymerase chain reaction
PI: Principal investigator
PTGR: Perceived threat of the risk of graft rejection
SARS-CoV-2: Severe acute respiratory syndrome coronavirus 2
sc: Subcutaneous
SOC: Standard of care
TB: Tuberculosis
TCZ: Tocilizumab
UACR: Urine albumin/creatine ratio
