# Supplementary material for: Tocilizumab in chronic active antibody-mediated rejection: rationale and protocol of an in-progress randomized controlled open-label multi-center trial (INTERCEPT study)
Source: Trials. 2024 Mar 22;25:213. doi: 10.1186/s13063-024-08020-0 (PMC10958896; doi:10.1186/s13063-024-08020-0)
Supplement: Supplementary file 1 — Additional file 1. Statistical analysis. [file 13063_2024_8020_MOESM1_ESM.docx]

**Statistical Analysis Plan**

***Screening Population***

The screened population will include all patients who have signed part A of the informed consent form (ICF) at visit 1 and have undergone screening tests. No analysis will be performed on this population, but only the number of patients screened will be reported.

***Randomized Population***

The randomized population will include all patients who have signed part B of the ICF at Visit 2 and are assigned a randomization number at Visit 2. No analysis will be performed on this population, but only the number of patients randomized will be reported.

***Intent-to-Treat Population***

The Intent-to-Treat (ITT) population will consist of all randomized patients who take at least one dose of assigned treatment and have at least one follow-up with measurements. The analysis of all efficacy data will be performed on the ITT population.

***Per-Protocol Population***

The per-protocol (PP) population will include all ITT patients without any major protocol deviations, who did not have to stop the study drug for > 4 weeks due to side-effects, and who had ≥80% compliance with study drug (with dose adjustments if required) while on treatment (up to discontinuation for patients whose treatment is terminated early). The PP analysis will be used to assess the robustness of the ITT analysis results of primary and secondary efficacy data.

***Safety Population***

All safety analyses will be conducted based on the safety population, which is defined as all randomized patients who receive at least one dose of study drug.

***Statistical methods***

The primary analysis of estimated glomerular filtration rate (eGFR) decline by therapy will be performed using a repeated measures linear model (two sided at alpha 5%) adjusting for eGFR at baseline and donor status (living versus deceased) as fixed effects and centre as random effect. Visits/time will be included in the model as a linear variable and the p-value of the interaction term of decline in eGFR and visit/time will be the primary analysis. The estimated treatment difference with the associated 95% confidence interval (CI) and P-value will be presented. Two-sided P-value less than 0.05 will be considered significant. The primary efficacy analysis will be performed on the ITT population and the robustness of the analysis will be performed on the PP population.

The hierarchical testing procedure below is introduced to guarantee that the probability of Type I error is < 5% for all confirmative statements. The order of the hierarchical testing procedure will be:

1. eGFR decline (Primary efficacy analysis, at 24 months)

2. Change from baseline in mean composite iBox risk prediction score at 24 months comparison between treatment groups

3. Change from baseline in immunodominant donor specific antibodies (iDSA)/mean fluorescence intensity (MFI) at 24 months comparison between treatment groups

4. Histologic response at 24 months comparison between treatment groups

5. Changes from baseline in proteinuria (urine albumin creatinine ratio, UACR continuous) at 24 months comparison between treatment groups

6. Incidence of patient survival up to 24 months comparison between treatment groups

7. Incidence of death-censored graft survival up to 24 months comparison between treatment groups

If the first analysis is significant the probability mass 0.05 will go to the second analysis and so on. If the first analysis is non-significant no analysis will be confirmative.

In the hierarchical testing procedure missing data will be imputed using multiple imputation (m=20) using relevant baseline covariates (age, sex, ethnic origin, weight, height, BMI, pulse, blood pressure (systolic and diastolic), body temperature and oxygen saturation) and follow up data (variables used in the hierarchical testing procedure) in the imputation process. In all the remaining tabulations and testing no imputations will be done. Analyses of the endpoint in the hierarchical testing procedure will also be performed without any imputations and then serve as sensitivity analyses.

Results from the other endpoints and from subgroups should be considered hypothesis generating only. P-values for the other endpoints than the eGFR (primary) and iBox, if presented, should be interpreted in a descriptive fashion only and cannot be considered as significant.

Other secondary endpoints apart from the ones in the hierarchical testing will be analyzed in an exploratory manner, using appropriate parametric and non-parametric statistical methods. For comparisons between two groups Fisher´s exact test will be used for binary response data, Mantel-Haenszel chi square test for ordered categorical data, the t-test for independent samples or Mann-Whitney U test for continuous data.

Continuous data will be expressed using mean (standard deviation) or median (interquartile range) and categorical data as numbers (frequencies). 95% confidence intervals will be calculated when appropriate. The results from the primary and secondary endpoints will also be stratified and presented according to sex/gender.

Graft survival, all-cause mortality and both adverse events (AE)/serious adverse events (SAE) event- free survival will be analyzed using Cox proportional hazards models presenting events per patient years, hazard ratio with 95% CI and KM-estimates over time including log-rank test.

If DSA (MFI) is found to be a predictor of rejection, receiver operative characteristic (ROC) curves will be used to determine the best cut- off of DSA level for the prediction of rejection.

For the qualitative research questionnaires, the frequency of answers per item as well as the general score (treated as a continuous variable) will be summarized per treatment arm. When comparing ordered categorical items between the groups, Mantel-Haenszel Chi Square test will be used. When comparing dichotomous answers (yes/no), Fisher’s exact test will be used. The sum scores will be tested using t‐test and Mann Whitney U test to evaluate differences between the groups. Correlation between the sum scores will be done by Spearman's rho test and graphically presented in scatter plots.

The secondary efficacy analysis will be performed both on the ITT and the PP populations.

Continuous data will be expressed using mean (standard deviation) or median (interquartile range) and categorical data as numbers (frequencies). 95% confidence intervals will be calculated when appropriate.

The results from the primary and secondary endpoints will also be stratified and presented according to sex. The number of AE/SAE will be summarized per category and treatment arm.

The statistical analysis will be performed using the commercially available software SAS v9.4. A detailed statistical analysis plan will be written where all populations, variables, and statistical methods will be described in detail. A qualified experienced statistician from the University of Gothenburg is responsible for the statistical analysis plan.

***Dropouts***

The total number of 50 subjects in the study (25 per arm) considers a total dropout rate of approximately 10%. Those patients in whom it will not be possible to measure the primary outcome at 24 months will be considered dropouts. These patients will still be included in the final ITT analysis. Thus, it is not anticipated that subjects who discontinue the study will need to be replaced in order to achieve the desired number of included subjects.

For the patients who drop out, their data will be analyzed up until the date of last available data.

Missing data will be analyzed with regards to reasons and pattern and sensitivity analyses will be performed based on various assumptions regarding the pattern.

Deviation from the original statistical analysis plan will be reported in the Clinical Study Report.

***Adjustment of significance and CI***

In this study, no other endpoints than the primary endpoint will be tested in a controlled fashion. It is therefore of importance to mention that the interpretation of results from the other endpoints and from subgroups should be considered hypothesis-generating only. P-values for the other endpoints than the primary, if presented, should be interpreted in a descriptive fashion only and cannot be considered as significant. The primary variable will be tested on 0.05 level and will be adjusted for eGFR at baseline and donor status (living versus deceased).

***Sample size calculations***

The sample size calculation is based on our preliminary analysis of chronic active antibody-mediated rejection (cAMR) patients at the Sahlgrenska University Hospital (SU) and assumption of an initial mean eGFR of 48±15 ml/min, and a mean decline of -7.5 ml/year

and an intra-patient correlation of 0.85 assuming a standard deviation of 15 throughout the visits. To uncover a difference of 10 ml (5ml/year) in eGFR slope at 24 months in the two arms, the calculated sample size based on eGFR measurements every 3 months using the above specified repeated measures linear model would be 25 patients in each arm, including dropouts (10%), using 1000 simulations, with a power of 80% at a significance level alpha of 0.05. The sample size is based on decline in eGFR only and therefore, no corrections for multiple comparisons are required.

According to current feasibility judgement, the aim is to recruit approximately 50% patients from the SU (Gothenburg and the regional outpatient clinics in Region Västra Gotaland), 35% from Karolinska hospital (KS, Stockholm and their regional outpatient clinics) and the remaining 15% from Akademiska hospital (AS, Uppsala and their regional outpatient clinics) as per the proportion of kidney transplantations performed at these centers.
